# Status and Challenges of Public Health Emergency Management in China Related to COVID-19

**DOI:** 10.3389/fpubh.2020.00250

**Published:** 2020-05-29

**Authors:** Yulong Cao, Jiao Shan, Zhizhong Gong, Jiqiu Kuang, Yan Gao

**Affiliations:** ^1^Department of Hospital-Acquired Infection Control, Peking University People's Hospital, Beijing, China; ^2^Department of Hospital-Acquired Infection Control, Beijing Jishuitan Hospital, Beijing, China; ^3^School of Public Policy & Management, Tsinghua University, Beijing, China

**Keywords:** public health emergency, emergency management, health policy, COVID-19, modernization

## Abstract

**Objective:** This study aimed at exploring the current development status and problems of health emergency management in China and provides a reference for improving, constructing, and implementing a public health emergency management system.

**Methods:** Cases of major and severe public health emergencies in China were analyzed along with the relevant health emergency management literature from the last decade.

**Results:** China's health emergency system gradually improved during the study period. Monitoring and early warning systems were significantly strengthened. Material reserves and transfer management systems were constantly improved. However, the operational efficiency of command and decision systems was low, versatile talent accounted for a relatively small proportion, and emergency fund investment was insufficient.

**Conclusion:** Constructing a sound and scientific emergency management mechanism is a lengthy and challenging process. To establish an emergency management mode for public health emergencies that is appropriate for China, it is necessary to solve existing problems and learn from the models and experiences of developed foreign countries.

In December 2019, a cluster of cases of severe acute respiratory syndrome coronavirus 2 (SARS-CoV-2) pneumonia emerged in Wuhan, Hubei, China. Although the cases were originally associated with exposure to the Huanan Seafood Market, current epidemiologic data indicate that person-to-person transmission of SARS-CoV-2 is occurring (1). As of May 1, 2020, a total of 3344,435 cases had been reported, including 238,788 deaths (2). This might only be the tip of the iceberg, with potentially more novel and severe zoonotic events on the horizon (3). SARS-CoV-2 has propagated in more than two hundred countries around the world, causing serious damage to human health and creating burdens for families, healthcare systems, and societies.

In the context of rapid development, such as global, economic, and information integration, China has experienced many acute infectious disease emergencies, including Severe Acute Respiratory Syndrome (SARS) in 2003, the H1N1 flu epidemic in 2009, the H7N9 avian flu epidemic in 2013, Middle East Respiratory Syndrome (MERS) in 2015, and, now, coronavirus disease 2019 (COVID-19). These epidemic events have had the systemic characteristics of sudden onset, diverse causes, widespread infection, unpredictability, serious, harmful consequences, and difficult management. Such events can have wide-ranging adverse effects on individual health, property, society, and the economy, posing serious threats to the overall well-being of a country (4).

On February 10, COVID-19 epidemic prevention and control was at a critical moment. The president of China, Xi Jinping, remarked while inspecting COVID-19 prevention and control work in Beijing that the COVID-19 epidemic is a major test for the country's emergency response capability. It demonstrates the advantages of China's system but also reveals weaknesses in the emergency response system, he said, adding that fundamental efforts should be made to intensify the modernization of the system and enhance the training of personnel (5).

Assessing impacts and strengthening emergency management have become the top priorities for the Chinese government (6). Therefore, this study aimed to appraise the current status of health emergency management in China and summarize its shortcomings and challenges. The findings can provide a reference for the government and related health institutions in improving the construction of China's health emergency management system.

The current status and progress of China's public health emergency were analyzed and summarized by searching the cases of severe and major public health emergencies and related health emergency management literature over the last decade.

## Development Status of Health Emergency Management

### Construction of the Health Emergency System and Improvements in Laws and Regulations

In the past, China's public health legislation was not proactive, and many laws were established retroactively. Public health legislation, government policy, and regulations were only established after major public health problems. In 1989, after the hepatitis A epidemic in Shanghai in 1988, the Contagious Diseases Act came into being. The Blood Donation Act was born in the early 1990s after a rise in HIV infection caused by paid donors. Since the beginning of the 21st century, many researchers have studied the impact of the model of American public health law on Chinese legislation (7). The Chinese government has gradually attached importance to and changed attitudes regarding health emergency management, made timely responses to major public health emergencies, and improved relevant laws and regulations. At the same time, the pace of emergency management system construction has gradually accelerated (8). Establishing an efficient and mature health emergency management system and scientifically regulating the handling of public health emergencies have become important parts of building a public health emergency response system. After the SARS outbreak in 2003, the Chinese Government heavily emphasized emergency preparation and related legislation. The government issued the “Regulations on Public Health Emergencies,” the “National Emergency Plan for Public Health Emergencies,” the “(Draft) Catalog of Health Emergency Personnel and Equipment,” and other documents. Through ongoing efforts, China established a system with general procedures, legal norms, and action plans for health emergency management. Since 2007, the core framework of “one case, three systems” has gone through four phases: planning, system construction, mechanism building, and legal construction. Moreover, it has established 25 sets of special plans and more than 80 projects (9).

The emergency experiences and practical lessons of the past 10 years have caused China's laws and regulations related to health emergencies to become self-contained (10). As of January 2019, 32 local laws and regulations with public health as the “target” were promulgated in all regions of China, which still have legal effect, showing the characteristics of: diversity in legislative subject, with a large time span, purpose and content of legislation are simple. Although the above-mentioned legislation guarantees the orderly development of local public health undertakings to a certain extent, it goes without saying that there are still some problems, such as difficulties in meeting the development needs of local public health undertakings in the new era, inadequate rigorousness of some legislative provisions, and poor operability. During the COVID-19 epidemic, we should clarify the legislative orientation and content and speed up the pace and process of local public health legislation. These changes will improve the quality and effectiveness of local public health legislation in China (11).

### Improved Monitoring and Early Warning Systems

Surveillance systems are an important source for early warning. Many countries have established such systems in order to be able to assess and control public health emergency events. Using the lessons learned from the SARS outbreaks, to address the threats of public health emergency events, China established the National Notifiable Infectious Disease Surveillance System, the Public Health Emergency Event Surveillance System, and the China Infectious Disease Automated-alert and Response System (CIDARS) (12). These systems are four-level from the national to the county and include all health care institutions across the country, allowing for the development and application of an early warning system at the county level in China (13). Some studies have compared the Chinese emergency events surveillance system with those of other countries and have found that China has a broader “all-hazard” approach, including, for example, chemical incidents (14).

All notifiable infectious disease cases should be reported in real time directly from hospitals via the Internet, and serious and unknown-cause infectious disease, such as plague, cholera, and COVID-19 must be reported to professional agencies designated by health administrative authorities within 2 h via telephone or fax, significantly increasing the surveillance timeliness for infectious diseases (15). The Chinese Center for Disease Control and Prevention analyzed the performance of CIDARS in 2016. The results show that a total of 325,208 signals were generated nationwide by the system, in which 323,271 (99.40%) were responded to, and 300,614 (92.44%) were responded to within 24 h. The median interval of the response time by different detection methods was 0.72–0.99 h (16).

Using technology to comprehensively integrate indicator-based, event-based, and syndromic surveillance systems has strengthened the detection of infectious diseases in China at all levels (17). China has achieved a phasic victory against COVID-19, but the epidemic situation of COVID-19 is still dangerous and complex. The use of digital technologies such as big data, artificial intelligence, and cloud computing must be encouraged so that they can serve as a pillar in the monitoring and analysis of outbreaks, virus tracing, epidemic control and prevention, medical treatment, and distribution of resources (18).

### Continuous Improvement of the Emergency Material Management System

The emergency medical supplies stored by health administration departments or hospitals determine the adequacy of such supplies, the efficiency of on-site emergency response, the proportion of casualties, the resulting economic losses, and the overall success or failure of emergency response (19). Since the SARS outbreak in 2003, a number of public health events have generated a great demand for emergency supplies in a short time, exposing a shortage of personal protective equipment reserves. This prompted the Chinese government at all levels to realize the necessity to reserve medical supplies for public health emergencies and gradually establish a reserve medical supplies system. As a result, China's reserve medical supplies system for public health emergencies has been continuously improved and developed (20).

COVID-19 became embedded in the population with great rapidity, spreading fast and infecting asymptomatically, even during the incubation period. It struck at the perfect time—right before China's most celebrated festival, which features the largest annual migration. As the pandemic has developed, a problem has become apparent: hospitals across the country have cited a vast shortage of medical supplies, especially personal protective supplies such as medical protective clothing and N95 masks; the hospitals are urgently calling for societal support (21). On January 23, the Ministry of Industry and Information Technology of China expedited the delivery of 10,000 sets of protective clothing and 50,000 sets of gloves to Wuhan from the National Medicines Reserve and established a national temporary production scheduling system for key enterprises and national temporary reserve supplies for epidemic prevention and control (22). China had set up a team to ensure medical supplies under the State Council that is responsible for the joint prevention and control mechanism of the COVID-19 epidemic. The joint prevention and control mechanism ensured the supply of medical equipment, materials, reagent test kits, and medicines, dealt with the epidemic at an early date, and helped to safeguard regional and global public health security.

To sum up, establishing an emergency medical supplies system is a material basis for improving the ability to respond to public health emergencies. It is an important guarantee for improving the comprehensive level of emergency management and for building a modernizing management system to respond more effectively to future infectious disease outbreaks.

## Problems and Challenges Facing Health Emergency Management

Over the last 20 years, China's health emergency management assessment system has experienced many rigorous tests when faced with a series of public health emergencies and has accumulated experience in detecting health emergencies and managing the weaknesses of the evaluation system.

### Need for Improvement in the Operational Efficiency of the Health Emergency Command and Decision System

A well-defined operational framework is an important subject in the management of public health emergencies, as it is responsible for good or bad performance and will hence decide the result of a public health emergency. Looking back at major epidemics in China, China urgently needs to improve its operational system for health emergency command and decision-making. In handling health emergencies, only a temporary emergency outbreak command center was established, which allowed temporary commanders to make emergency decisions and arrangements. As a result, the government was absent from the deployment of health emergency agencies, disease control centers, and related departments, resulting in poor emergency coordination among relevant departments. This severely affected the efficiency of emergency decision-making and disposal (23). Based on 259 public-health studies published in China from 2003 to 2013, Liu et al. identified 31 problems with the government's emergency responses to health emergencies, finding that “poor collaboration between health emergency management departments” is an issue that requires particular attention (24).

Due to geographical differences, uneven economic development, and different policy support, there is variation in conditions in different jurisdictions, presenting very substantial challenges to health emergency management and militating against adopting one-size-fits-all policy solutions (25). Investigating Guangdong's health emergency command system from 2015 to 2016, Huang et al. found that it adopted advanced communication technology, using multi-person telephone discussions and replacing the common fax machine with the electronic fax machine, and that it generally performed better than such systems in other provinces, basically meeting the local needs. Functional modules of decision analysis still needed to be improved, as this deficiency restricted the efficiency of health emergency personnel and departments (26).

Although China established a quasi-wartime work mechanism led by the country's top leader after the epidemic broke out, for administrative health departments and emergency response agencies to achieve efficient and standardized operation, coordination, and disposal, it is necessary to improve and optimize the health emergency command and decision system. The Chinese government responded with determination, and therefore the success in controlling the epidemic nationally may hold useful lessons for other public health services around the world. What is needed in the connected global community is mutual support and cross-border multi-sectoral collaboration. Only when there is trust, cooperation, and understanding among governments, prevention and control agencies, and health emergency agencies, can emergencies be handled efficiently (27).

### Lack of Emergency Professionals

China has been deeply affected by the trend toward multifactor public health emergencies. Talents with rich theoretical knowledge and practical abilities have played an important role in the healthcare system. A phenomenon of the “false saturation” of talent has emerged in China's public health system, that is, all-round professional emergency personnel with solid theoretical knowledge and rich practical experience capabilities are scarce (28). A national health service system planning outline (2015–2020) was put forward that stated that, by 2020, China would have 0.83 public health personnel per 1,000 permanent residents, but in 2017, there were only 0.61 public health workers per 1,000 permanent residents (29). According to the 2018 China Health Statistical Yearbook, on personnel size, there were only 114,000 public health doctors, accounting for only 3% of the total medical practitioners, far less than the number of oral physicians (217,000), traditional Chinese medical doctors (576,000), and clinicians (2.7 million). On educational structure, more than half (54%) of the personnel in China's centers for disease control and prevention at all levels had only a college degree, about one-third (37%) had a bachelor's degree, and only 7% had a master's degree. The statistics show that from 2009 to 2017, despite an increase of 76.3% in the number of health personnel in hospitals, the number of staff in disease control and prevention institutions decreased, and the number of disease control staff and health technicians staff decreased by 3.0 percent and 4.1 percent, respectively (30). In addition, poor career preparation and low funding for personnel training inhibit the improvement and development of health emergency capabilities (31). According to the latest data from the Association of Schools and Programs of Public Health (ASPPH), 61,453 public health students were trained by accredited institutions in 2018, of whom 37% were undergraduates, 49% were masters students, and 14% were doctoral students (32). There is still a long way to go in the training of public health talents in China.

### Insufficient Emergency Funding

Public health services are regarded as a public welfare undertaking provided by the government to all of the residents; they play a vital role in the prevention and control of various diseases (33). At the same time, the adequacy of the government investment in public health emergency funds affects the health emergency management mechanism to a certain extent and also plays a key role in the equalization of basic medical and health services in China (34).

The public health emergency management systems in developed countries are worth using as references for emergency systems reform in China. The annual budget for public health emergencies in the United States exceeds $12 billion. Statistics show that the US CDC allocates billions of dollars (60% of its total budget) to state and municipal health agencies each year. In addition to the budget itself, the projects and funds of international organizations are included in the CDC work plan, which generally exceeds more than double the budget (35). The European Union established the European CDC (ECDC) in Stockholm, Sweden, in 2005. Its main task is to strengthen the sharing and coordination of health resources and to unify the EU's disease control work. Each member country of the ECDC has a special coordination agency to realize the sharing of public health information and resources among European countries to jointly respond to various emergencies and epidemics. The budget for the ECDC was put at ~60 million euros for 2014 (36).

Underfunded by the government, some county-level health bureaus and CDCs in China have never received health emergency subsidies from the government. This can be seen from government financial input in the 5 years from 2014 to 2019. In 2014, the state allocated 529 million yuan for the “Special funds for public health emergency,” but by 2019, the government rolled back its investment to 450 million yuan, down 14.9 percent from the previous year. In contrast, the fiscal allocation for public hospitals in 2014 was 3.619 billion yuan, which increased to 5.023 billion yuan by 2019, a year-on-year increase of 38.8% (37). These phenomena lead to a reduction in the financial security capacity of some less developed areas in China, which has made it difficult to implement expenditures in these areas in terms of monitoring and early warning, emergency drills, and campaign promotion.

With China's rapid development, response to health emergencies faces various challenges. China has been gradually improving its health-emergency-related surveillance, plans, mechanisms, legal systems, equipment, and guarantees. As a result, China has been able to monitor and prepare for health emergencies, prevent epidemics, and deal with on-site disposal and reconstruction after disasters. However, it must also be acknowledged that China's health emergency management work started relatively late and is immature, thus leading to deficiencies in some areas, such as government funding, talent training, and public health communication. Therefore, China should learn from the emergency-management experiences and the models of developed foreign countries and adapt them to the Chinese context to form an effective and appropriate public health emergency management model for China. Overall, building a public health emergency response mechanism that is scientific and sound will be a long and complicated project.

## Author Contributions

YC: study design, data collection and writing. JS: data collection and writing. ZG: data collection. JK: study design and data analysis. YG: study design and writing.

## Conflict of Interest

The authors declare that the research was conducted in the absence of any commercial or financial relationships that could be construed as a potential conflict of interest.
